# Universal Recommendations on Planning and Performing the Auditory Brainstem Responses (ABR) with a Focus on Mice and Rats

**DOI:** 10.3390/audiolres13030039

**Published:** 2023-06-02

**Authors:** Ewa Domarecka, Agnieszka J. Szczepek

**Affiliations:** 1 Department of Otorhinolaryngology, Head and Neck Surgery, Charité-Universitätsmedizin Berlin, Corporate Member of Freie Universität Berlin and Humboldt Universität zu Berlin, 10117 Berlin, Germany; 2 Faculty of Medicine and Health Sciences, University of Zielona Gora, 65-046 Zielona Gora, Poland

**Keywords:** auditory brainstem responses, ABR, translational audiology, experimental audiology, rodents

## Abstract

Translational audiology research aims to transfer basic research findings into practical clinical applications. While animal studies provide essential knowledge for translational research, there is an urgent need to improve the reproducibility of data derived from these studies. Sources of variability in animal research can be grouped into three areas: animal, equipment, and experimental. To increase standardization in animal research, we developed universal recommendations for designing and conducting studies using a standard audiological method: auditory brainstem response (ABR). The recommendations are domain-specific and are intended to guide the reader through the issues that are important when applying for ABR approval, preparing for, and conducting ABR experiments. Better experimental standardization, which is the goal of these guidelines, is expected to improve the understanding and interpretation of results, reduce the number of animals used in preclinical studies, and improve the translation of knowledge to the clinic.

## 1. Introduction

Translational research aims to apply basic research findings to clinical practice. A translational project in audiology may involve pharmacological research, the development of non-pharmacological therapies, or disease monitoring [1]. The auditory brainstem response (ABR), also known as brainstem auditory evoked potentials (BAEPs) and short-latency auditory evoked potentials (SLAEPs), is a sensitive tool for determining the therapeutic potential of hearing loss therapies [2,3,4,5] and for diagnosing auditory nerve and brainstem dysfunction. Because of its objective nature, ABR is one of the few audiological tests that can be used for both human diagnostics and animal research. The results obtained from animal studies have translational potential and represent an essential step in developing therapeutics for otological disorders. According to the classification of translational research, such studies represent translational steps T0 (basic research aimed at understanding the pathological mechanisms of hearing loss or developing curative approaches) and reverse translation T1 (bedside to bench).

During an ABR, the electrical activity of auditory fibers evoked by an acoustic stimulus is recorded by electrodes placed on the skin (in humans) or subcutaneously (in animals) near the ear and auditory brainstem. ABRs consist of up to seven positive peaks, or waves, numbered from I to VII. In humans, wave I represents cochlear nerve activity (compound action potential, CAP), and wave II marks the exit of the cochlear nerve from the skull at the temporal bone. Waves III–V represent auditory brainstem activity [6]. Two key features of the ABR waves are their amplitudes and latencies. The amplitudes of the ABR waves provide information about the degree of synchronous action potential and the natural generators or modulators of the signal [6,7]. ABR latency reflects axonal conduction time and synaptic delay [6,8]. In clinical practice, ABR may help detect auditory neuropathies, retrocochlear lesions, or vestibular schwannomas [9]. It is also used for intraoperative monitoring to determine cochlear implant performance [10].

**Figure 1:**
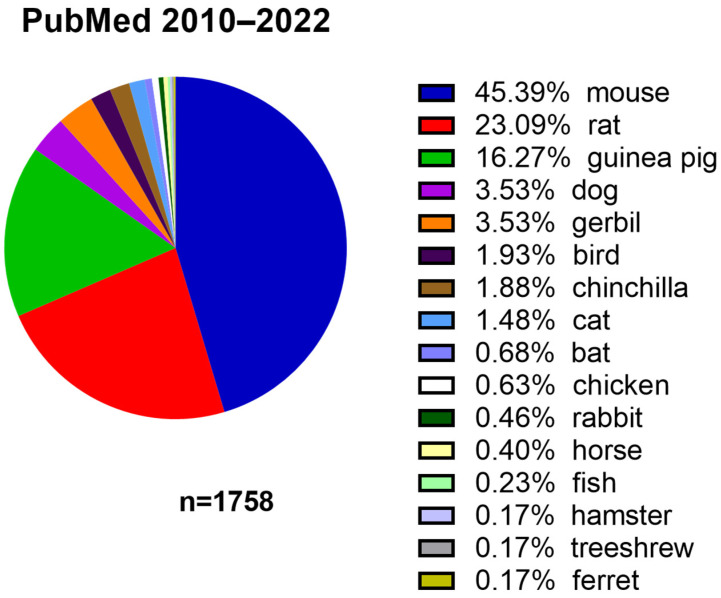
Usage of animal species in studies involving ABR published in PubMed between 2010 and 2022.

A great deal of insight into the development of ABR in animals has come from research on cats, and cats were the first group of animals in which ABR developmental changes were determined [11,12,13]. Depending on the research question, different animals, such as chickens, chinchillas, dogs, and bats, are used in experimental audiology. However, this paper will primarily focus on rats and mice, the two species most commonly used to study auditory responses using ABR (Figure 1).

Rats are primarily used in pharmacological studies to develop new compounds [14]. Guinea pigs provide easy access to the cochlea and round window. Therefore, these animals are often used to research the round window approach of drug delivery and perilymph sampling [15]. Mice are used in genetic studies of inner ear pathology, although their hearing range differs from that of humans [16]. The gerbil’s longevity (approximately three years) and resistance to developing middle ear disease make it an excellent model for age-related hearing loss [17].

Despite anatomical differences in the origins of ABR waves between species [18], the amplitude of wave I, which reflects the functional status of cochlear ribbon synapses and represents the functional integrity of auditory nerve fibers [19,20], is a sensitive marker of synaptopathy (recorded to suprathreshold transients) in both humans [21] and animals [22]. Wave V in humans probably corresponds to wave IV in animals [23].

**Figure 2:**
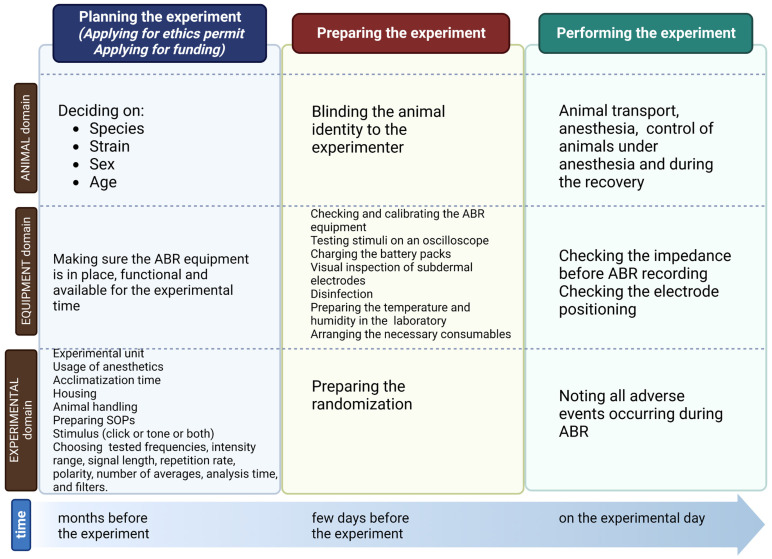
Schematic presentation of timelines and domains during the planning, preparation, and experimentation. Created with BioRender.com.

Experimental ABR studies often yield significant results of potential clinical significance. However, data heterogeneity often precludes translation to the clinic. Data heterogeneity is related to several factors representing three domains: animal-, equipment-, and experiment-dependent. In the previous work, we analyzed the impact of each of these domains on the results of the ABR [24,25]. Here, we synthesized the knowledge on performing ABR in experimental animals to provide general recommendations. These recommendations consist of three parts: the planning of the experiment, the preparation of the ABR recordings, and the performance of the ABR recordings concerning three domains: animal-, equipment-, and experiment-related (Figure 2). Improving the reliability of results and minimizing experimental variability are the ultimate goals of the recommendations [26].

## 2. Planning the Experiment

The Experimental Design section refers to the outline of the experiment, which is usually part of the application to the ethics committee and/or the research grant application. After approval by the authorities, the only way to change this plan is to submit a supplemental application. Therefore, it is imperative at this stage to think through the project, discuss the procedure with laboratory members, and, if necessary, with the local veterinarian or animal facility representative. The more details addressed at this stage, the more time and peace of mind can be gained in conducting the experiments.

Because each country has different laws regarding the use of animals in research, no documents or links are given here. The investigator should find out how, when, and how to apply for an animal license in their institution. The first step in performing ABR on any animal is to obtain an animal use permit.

### 2.1. Planning the Experiment: Animals

**Figure 3:**
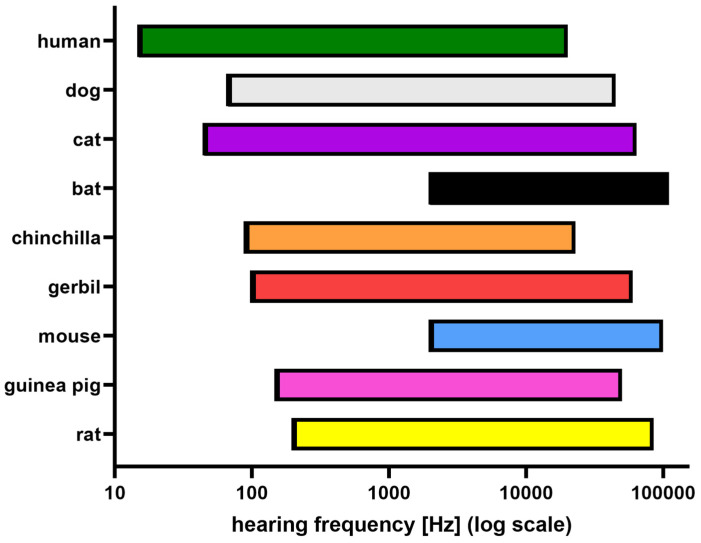
The hearing ranges of the most commonly used laboratory animals in ABR studies as compared to those of humans. Adapted from [28]. Due to the different paradigms that have been used to measure hearing range in different species, caution should be used in the interpretation of these results [29].

This section addresses the species, strain (albino or pigmented), sex, age of the animals, and the number of animals included in the planned study. The animal’s hearing range, size, and approximate life expectancy should be considered to select the appropriate species. The approximate hearing ranges of animals compared to humans are summarized in Figure 3. However, the choice of animal species may be influenced by factors other than hearing range, such as anatomical characteristics, life expectancy, or susceptibility to substances toxic to humans. For example, guinea pigs have larger tympanic bullae, which provide better access to the inner ear and are used in drug delivery studies by injection through the tympanic membrane or semicircular canal into the inner ear or for performing inner ear surgery. Due to their long lifespan (up to 20 years), chinchillas are not a standard model for age-related hearing loss [17], despite their hearing range being similar to humans. In the study of drug-induced hearing loss, a variety of species are used, and the different susceptibility of the species to ototoxins necessitates different dosage regimens [27]. Compared to guinea pigs, rats and mice are less susceptible to aminoglycoside-induced ototoxicity.

Both ABR thresholds and waveforms reflect strain differences [30,31]. For example, adult male (3–6 months old) Sprague-Dawley rats have a lower hearing threshold than Long-Evans rats (2–8 kHz). Adult (8-week) female Sprague-Dawley and Wistar rats had lower hearing thresholds below 26 kHz than Long-Evans and Lister Hooded rats [30]. Differences were observed between Sprague-Dawley and Wistar rats in the amplitude of wave IV: Sprague-Dawley rats had a higher amplitude than Wistars. Amplitude differences between Sprague-Dawley and Long-Evans strains were only observed when an 8 kHz tone burst elicited the ABR response. Sprague-Dawley rats have a higher amplitude of waves II, III, and IV than the Long-Evans strain [31]. Waves VI and VII are absent in animals [23].

Concerning laboratory mice, strain-dependent differences in the onset of age-related hearing loss (ARHL) have been reported (Table 1). Because CBA/CaJ mice have stable hearing (until 12–18 months of age), they are used in chronic ototoxic exposure experiments [32]. See elsewhere for more details on ARHL in mice [33].

Interestingly, unpigmented and pigmented animals have different inner ear morphologies [37,38,39,40]. Furthermore, melanin protected guinea pigs from noise-induced hearing loss [41]. Melanin precursors protected albino mice from age- and noise-induced hearing loss [42]. In addition, it was observed that the onset of age-related hearing loss differed between wild-type C57BL/6 and C57BL/6 Tyrc-2J albino mice, which was attributed to a melanin-dependent thinning of the striae, marginal cell loss, and a reduction of endocochlear potential [43].

There are behavioral differences between the strains of animals at the beginning and after the experimental treatment. Strain differences have been observed in male mice for sheltering behavior, locomotor activity, and behavior related to the dark/light phase [44]. Female mice from different strains have different sleeping habits [45]. Following noise exposure and salicylate administration, male Wistar rats developed more aggressive behavior than Sprague-Dawley rats [46]. Sometimes only female mice are used in a study because aggressive behavior may occur in large groups of unfamiliar male mice [47].

Sex bias has been identified as one of the factors contributing to poor translation in preclinical research [48]. Sex should be treated as a biological variable, and both sexes (equal numbers) should be included in the study design [48]. Exceptions to this are studies on the prevalence of the disease in only one sex, the performance of confirmatory experiments, or a pilot study. The justification should be provided in the study design if animals of only one sex are used. In audiology research, the effect of sex is reflected in differences in hearing ability [49], metabolism, and efficacy of medications [50]. Since the menstrual cycle affects the hearing thresholds of women, it should also be considered a confounding factor in animal studies [51]. Although sex affects the onset of presbyacusis, and male Fischer 344 rats exhibited age-related hearing loss earlier than female rats [52], differences in ABR latencies in the aging cochlea of CBA and C57 mice in males and females were not detected, which was attributed to minimal differences in brain size between the sexes [53]. Additionally, body mass and head/neck fat layer are confounding factors, as both are sex- and age-dependent [54,55]. Compared to females, male CBA/Ca mice are more susceptible to the adverse effects of a high-fat diet on body weight, metabolism, and hearing [56]. Because the subcutaneous fat layer has high electrical resistance and low conductance properties, and skin and muscle have low resistance and high conductance, this can result in high electrode impedance and affect ABR results [57].

The susceptibility of mice to noise and drug-induced hearing loss is age dependent. The noise susceptibility window in CBA/J mice begins at fifteen days of age and remains high until three months [58,59]. Consistent with these findings, young adult (1–2 months old) C57Blk/6J, CBA/CaJ, and Balb/CJ mice were more likely to develop noise-induced permanent threshold shifts than 5–7 month-old mice [60]. Similarly, susceptibility to ototoxic damage is age-dependent, and mice are most sensitive to drugs such as kanamycin during the first month of life [61,62]. Furthermore, susceptibility to drug-induced hearing loss depends on the exposure time and dose [63,64,65].

An essential step in preparing the experimental design is to decide how many animals will be included in each experimental group. A power analysis calculation should be performed to determine the sample size [66]. This mandatory calculation requires knowledge of effect size (significant difference between groups), standard deviation (only used for quantitative variables), power (probability of finding), the direction of effect, statistical test (simple vs. complex tests), and expected attrition of animal deaths [67]. An alternative method of sample size calculation is based on the law of diminishing returns—a technique used when it is difficult to specify an effect size [68]. The number of animals used in experiments should be kept as low as possible for ethical and practical reasons. Typically, 5–10 animals per group are used, which may not be sufficient for statistical analysis [69]. In such a case, a solution may be to perform pilot studies, sometimes with only one animal per group [66]. According to the ARRIVE guidelines, a justification for the number of animals included in the study should be reported.

Inclusion and exclusion criteria should be decided and not changed during the experiment. Examples of universal exclusion criteria include general animal health (abnormal appearance, tumors, otitis media, redness and swelling of local tissues, perforated tympanic membranes) and animal distress (appearance, behavior).

Finally, it is recommended that a Standard Operating Procedure (SOP) be prepared in the event of infectious contamination. Since the treatment given to one animal may affect other animals in the cage, it is good practice to consider all such animals as treated and include them in the same experimental unit.

### 2.2. Planning the Experiment: Equipment

An essential step is to ensure that the ABR equipment is in place, functional, and available for the duration of the experiment. There are several commercially available systems used in animal audiometry, manufactured by (in alphabetical order): ADInstruments (Castle Hill, Australia); Intelligent Hearing Systems (IHS, Miami, FL, USA); Interacoustic (Middelfart, Denmark); Neuro-Audio 0710 (Ivanovo, Russia); and Tucker Davis Technologies (TDT, Miami, FL, USA) [24]. Future studies should examine whether ABR results differ by device. Manufacturer’s instructions for calibration and oscilloscope signal testing should be followed.

### 2.3. Planning the Experiment: Experiment

This domain includes stimulus design, the experimental unit, anesthetic use, acclimation time, housing, animal handling, and the possible influence of stress.

Although different commercial systems are used to perform small animal audiometry, the same stimulus parameters should be used for evoked ABR. The detailed protocol for performing ABR with TDT equipment and the IHS system [70] has been described elsewhere [71]. A video protocol showing ABR measurements in mice has also been described [72].

Depending on the purpose of the study, click or pure tone stimuli are used to elicit the ABR: tone burst stimuli are used to assess frequency-specific hearing, whereas click is used to assess high-frequency hearing, diagnose auditory nervous system disorders, or rapidly screen for hearing loss. The time required to complete the audiologic measurement is affected by either click or a tone burst.

The auditory stimulus consists of a sound spectrum, an intensity range, a signal length, a repetition rate, and a polarity. In addition, a number of averages, analysis time, and filters must be defined. Both the stimulus (e.g., type of stimulus, polarity) and the acquisition parameters (e.g., filters, analysis time) play a critical role in the quality of the ABR recording. Their effects are summarized in Table 2.

Either the entire hearing range or only a few selected frequencies are tested. The frequencies tested depend on the species. In mice and rats, typical test ranges include 4 kHz to 32 kHz, whereas, in guinea pigs, they range from 1 kHz to 18 kHz [73]. For gerbils, test ranges include 1 kHz to 8 kHz [74].

The click stimulus (a broadband signal) is characterized by a rapid onset and short duration. It activates more auditory nerve fibers and produces larger ABR amplitudes because its energy spans a broader frequency range than a tone burst [6]. The stimulus level can be independently adjusted. Three stimulus polarities are used: rarefaction, condensation, and alternating. Condensation clicks initially move the tympanic membrane inward, whereas rarefaction clicks move the tympanic membrane in the opposite direction [75]. The effect of the polarity of the ABR recordings has been discussed in the literature [23]. In humans with normal hearing, ABR recordings with either condensation or rarefaction polarity are nearly identical. Differences between click-evoked ABRs with condensing and rarefaction polarity were shown in cats [76]. For rarefaction clicks, the amplitude of wave I is greater. When recording is unreliable, alternating polarity (switching between condensation and rarefaction) can be used. Eliminating stimulus artifacts and cochlear microphonics makes Wave I more easily detectable [23].

The auditory stimulus can also be characterized by its repetition rate. As the stimulus repetition rate increases, amplitudes decrease, and latencies increase. Therefore, a slower stimulus rate will result in a more visible ABR waveform.

Windowing defines the shape of the tone burst signal, and it follows the rise, plateau, and fall of the stimulus. A stimulus that is too short will result in spectral splatter, while a stimulus that is too long will not produce a well-defined ABR (lower amplitude). Therefore, a 2-1-2 tone burst (two cycles of rise/fall time and one cycle of plateau time) is considered a compromise in humans [77]. In mice, 2.5 ms has been used in most studies [73].

In addition, noise reduction and averaging techniques are required for reliable amplitude assessment [78]. Noise can be environmental, instrumental, or generated within the body (physiological noise). Filters are used to improve the signal-to-noise ratio (SNR) [79]. A high-pass filter removes low-frequency noise, while a low-pass filter removes high-frequency noise [75]. Since electrophysiological signals are often plagued by noise from the power line (50 or 60 Hz and harmonics), a notch filter is used to eliminate this noise [80]. A detailed description of the use of filters has been provided by Cheveigné and Nelken [80]. Signal distribution/loudspeaker placement depends on the manufacturer’s recommendations.

Because decreasing stimulus intensity results in longer latencies and lower amplitudes of ABR waves, 2–3 ABR traces should be collected at a near-threshold intensity to estimate the ABR threshold and correctly identify relevant peaks. In addition, low amplitude responses require more averages to verify ABR results. Since the ABR signal typically occurs in the 1 to 2.5 microvolt range, amplification is needed. A gain is used to remove the effect of hardware amplification of the response signal [73]. Use the value specified by the manufacturer.

**Table 2:** Factors influencing ABR recordings.

Factor	Definition	Influence on ABR Results	Suggestions Based on ABR User Guide
Ramp Number of Cycles (Rise-Plateau-Fall, e.g., 5 ms (2-1-2))	The number of sinusoidal waves in the rise, plateau, and fall portions of the tone burst’s waveform. Only applicable for tone-burst	An increment in the rise time of the signal stimulus results in elongated absolute latencies [81]	mouse studies: mainly 2.5 ms
Repetition rate	Number of stimuli produced per second	Amplitude decrease with an increasing repetition rate of the stimuli—an increase in repetition rate results in an increase in ABR latencies.	21/s
Polarity	Crucial for initial stimulus presentation since it determines the way the sound pressure wave is presented [82]	Three stimulus polarities are used; i.e., rarefaction, condensation, and alternating. The latency of waves I, III, and V are shorter in response to the rarefaction click than the condensation click [83].	Rarefaction or alternating
Number of averages		Impact on the signal-to-noise ratio. The number of averages balances signal quality and minimalization of the time to complete testing.	The typical range of averages: 256–1024
Analysis time/Recording window	A period following the stimulus is presented to the subject, during which the response is averaged and analyzed	Since decreasing stimulus intensity reduces the amplitude and increases latencies, the analysis time is extended to 15 ms to estimate the hearing threshold.	10 ms
Sampling rate	The average number of samples acquired per second		12 KHz
Artifact Rejection Threshold	The value defines the lowest level of electrophysiological activity, which contains excessive electric noise.	Clearer ABR response	
Filters	Use filters to separate signals based on their frequency, attenuating (reducing in amplitude) the unwanted frequency components and/or emphasizing the features that are important to us [79]	Filters make the presence or absence of the ABR responses more obvious since noise is filtered out.	Highpass filter: 300 Hz Lowpass filter: 3 kHz

Three main study designs are used in experimental audiology. The first type uses one ear as the experimental ear, while the contralateral ear is the control. This model is predominantly used in studies of ear surgery or the efficacy of drug administration. To use this model in studies of noise-induced hearing loss, an earplug must be used to protect one ear during noise exposure, while in studies of drug-induced hearing loss, the drug should be locally rather than systemically administered. In the second type of study, animals are divided into experimental and control groups. In the third type of study, all animals are examined at baseline and after treatment (before-after). This model ensures better identification of changes in ABR thresholds and waveforms by eliminating inter-animal differences and controlling for baseline variations.

When the purpose of the study is to estimate the effect of earlier treatment on hearing function, to determine whether hearing changes are gradually developing, and finally to test whether these changes are transient or permanent, the time course of recovery of ABR results is analyzed. One of the ways to assess this is to calculate the correlation factor (corF), which reflects the changes in waveform and amplitude before and after exposure to noise or toxic substances. High values (around 1) indicate the similarity of a waveform, whereas low values (around 0) reflect the loss of both waveform similarity and amplitude [84,85]. Depending on the experiment’s length, the fat layer’s thickness and the animals’ age should be considered confounding factors.

Next, the number of animals to be tested daily with ABR should be determined. It is recommended to perform the audiological tests at the same time of the day to minimize the possible effects of diurnal rhythm since the function of the auditory system (both peripheral and central) is regulated by circadian mechanisms [86]. In addition, problems with anesthesia may prolong the measurement time.

The choice of an appropriate anesthetic is of great importance. Depending on laboratory capabilities and ethical requirements in a given country, inhalation gas or injectable drugs may be used. In the case of inhalation anesthesia, the anesthesia equipment should have been calibrated within the last 12 months. The local animal caretaker or veterinarian may be consulted for advice on the appropriate anesthetic and dosage. In most studies, isoflurane is used as an inhalation anesthetic, whereas ketamine/xylazine mixtures are used for injections. Technical details on the administration of isoflurane to animals can be found elsewhere [87]. Although the same drugs are used, different doses are applied, which affects the anesthetic conditions (working time and depth of anesthesia) [88] and explains the high heterogeneity of results. The advantages and disadvantages of using these anesthetics are summarized in Table 3.

Injectable anesthetics can be intraperitoneally, intramuscularly, or intravenously administered [93]. However, each type of injection requires proper restraint technique and has disadvantages, such as difficulty finding a superficial vein (intravenous), a high failure rate (intraperitoneal) [94,95], a less predictable route (subcutaneous) [96], or tissue reactions (intramuscular) [97]. Learning proper restraint techniques is recommended to avoid overdosing with injectable anesthetics. It is important to first assess the effects of anesthesia on an animal, to use healthy animals, to use drugs with a wide margin of safety, and to use comfortable syringes, such as those used to inject insulin in humans [98].

If ABR is to be recorded more than once, the SOP for recovery from anesthesia should be prepared.

At least one week of acclimatization should be allowed after transport to the animal facility to prevent the influence of stress on the experimental outcome. During this time, transport-induced metabolic and hormonal changes return to baseline [99]. The acclimation period should be extended if the day/night cycle is reversed.

The type and size of cages used should be appropriate to the number of animals housed per cage. Welfare assessments should be planned before, during, and after the experiment. The minimum cage size can be estimated based on the age of the animals when they are permanently removed from their home cage. Cage replacement should be avoided. If the animals are aggressive and must be separated from cage mates, the SOP should be prepared for such a situation.

An additional confounding factor in animal studies is stress, which has already been mentioned a number of times in this paper. Since both physical and psychological stress affect the hearing ability of animals [7], it is necessary to identify all possible stressors in the study design, such as environmental noise, handling, isolation, cage changes, and injections.

- Environmental noise can impact the hearing abilities of animals. Human activity is the primary source of environmental noise; therefore, all noise-generating activities inside the animal facility should be reduced to a minimum [100,101].
- The SOP describing the handling of animals should be prepared beforehand, and all unnecessary handling should be avoided. A handling tunnel or cupping without restraint in the open hand can minimize the anxiety of mice [102]. It is worth noting that the presence of men in breeding or experimental rooms is stressful for mice [103]. Animal behavior is also influenced by the animals’ familiarity with the personnel involved in the experiment [104]. Importantly, the same breeds of animals purchased from different suppliers may respond to stress in various ways [105].
- Note: Experimenters should not wear scented cosmetics [106].
- Since cage changing is stressful for animals, cleaning cages should be planned in advance [106].
- In addition, social isolation can cause somatic reactions and should be avoided.
- Repeated intraperitoneal injections are also known to stress animals. Attention task performance was similar in rats chronically sham injected and chronically sham injected and restrained [107].

It is important to dedicate a person responsible for feeding and observing animals during the experiment. Abnormal posture, changes in motoric activity, reduced water and food intake (differences in the size of animals are a source of variability between animals), gait disturbance, erected hairs, weight loss, hypersalivation, abnormal licking, chewing movement, tremor, desensitization, moaning, and aggressive behavior should be observed and marked in the experimental protocol.

## 3. Preparing ABR Recordings

### 3.1. Preparing ABR Recordings: Animals

The experimenter should be blinded to the animal’s identity (e.g., treated or untreated). Tips for improving study blinding have been described elsewhere [108]. Nevertheless, blinding (masking) is not always possible, e.g., when performing a comparative analysis of older and younger animals, as older animals are heavier and present alternation in body conditions compared to young animals. Based on ABR recording, noise-exposed animals are easily identified during the experiment [109].

### 3.2. Preparing ABR Recordings: Equipment

Two to three days before measurement, the system used to measure the ABR should be thoroughly checked and calibrated. Because calibration requires proper equipment, it should always be performed according to the manufacturer’s instructions. In addition, an oscilloscope test should be performed to provide a visual representation of the shape or waveform of the signal. Again, follow the manufacturer’s instructions.

Battery packs should be fully charged. Visual inspection of the subdermal electrodes should determine the need for replacement due to corrosion, blunting, bending, or other damage. The disinfectant should be prepared, and the experimental instruments should be disinfected or autoclaved. In addition, the environmental noise level should be determined by performing a saline test. See elsewhere for details [110].

The conditions (temperature, humidity) in the animal facility should be noted to mimic them as closely as possible in the experimental area (ABR room). The experimental area should be prepared to avoid unnecessary movements (gloves, syringes, etc., should be ready to be at hand).

### 3.3. Preparing ABR Recordings: Experiment

The random assignment of animals to experimental groups is necessary to minimize the effects of subjective bias. The PREPARE and ARRIVE guidelines identify randomization as mandatory in any animal study. Programs such as IBM SPSS Statistics, Prism GraphPad (www.graphpad.com/quickcalcs), randomizer.org, or the RandoMice tools can perform randomization [111]. Practical information on randomization can also be found on the ReproducibiliTeach YouTube channel (https://www.youtube.com/@reproducibiliteach, accessed on 19 April 2023).

## 4. Performing ABR

### 4.1. Performing ABR: Animals

This area covers animal transfer to the ABR research facility, anesthesia, and the monitoring of anesthetized animals.

After being transferred from the animal facility to the laboratory, animals should be allowed at least 15 min to acclimate. If possible, avoid changing cages during the transport and isolation of animals. Because small mammals (mice, rats, hamsters, guinea pigs, and rabbits) cannot regurgitate, it is not recommended that food be withheld before anesthesia—these animals should be provided with water and food ad libitum. However, in carnivores (e.g., dogs, cats, or ferrets) and insectivores (e.g., bats), food deprivation is mandatory as part of the preparation for anesthesia. Details can be found on the homepage of the Society for Laboratory Animal Science (https://www.gv-solas.de/?lang=en, accessed on 24 March 2023) or in a publication dedicated to that topic [112].

If injectable anesthesia is used, before removing the animal from the cage, it should be ensured that the animal is not exhibiting aggressive behavior. If so, wait until the animal has calmed down. Prepare an appropriate dose of anesthetic based on the animal’s weight in a sterile vial or bottle (shake well before use). Mixed drugs should be protected from light and stored at room temperature on an experimental day. The rest of the drug or drugs mix or their waste should be disposed of according to local regulations—it equally regards the inhalation and non-inhalation drugs [113].

The most consistent and artifact-free ABR signals are obtained from stable anesthetized animals; otherwise, spontaneous muscle twitches over 100 times larger than the ABR may occur and completely overwhelm the signal. Applying ophthalmic ointment to both eyes is recommended to reduce the risk of corneal abrasions. Susceptibility to corneal injury is strain dependent [90].

An awake animal should not be housed with an anesthetized animal [114]. Five minutes after applying anesthesia (on average, usual time between the injection and loss of motor responses to noxious stimuli), check whether the animal is deeply anesthetized (eyelid reflex, toe reflex, tail flick reflex, nose, and vibrissae). For details on the depth and stages of anesthesia, please refer to a specific publication [115]. If the reflexes are still present, wait another 5 min, and if deep anesthesia cannot be confirmed after this time, review the protocol and refer to the SOP. Further action (additional injections, calling a veterinarian, returning the animal to the facility, or sacrificing the animal) depends on the country, ethical approval, and specific local regulations.

Once the animal is under deep anesthesia, the skin can be disinfected, and the electrodes subdermally placed. Care should be taken to ensure the electrodes are always placed in the same position. If the recording is repeated (recovery experiments), the position of the electrodes should be marked (e.g., by shaving the areas).

Transfer the animal to the ABR room. Perform an otoscopy to check the condition of the middle ear. Animals with ear canal or tympanic membrane abnormalities should be excluded from further experiments [24]. If necessary, remove the earwax.

During the anesthesia, the animal should be covered (e.g., with paper towels—do not use electric heating pads as most of them may interfere with the ABR recording) to keep it warm. Monitor and maintain the animal’s body temperature to prevent the disruption of thermoregulation and eliminate the body temperature’s effect on ABR records. A temperature decrease of 0.5 °C or more may significantly alter ABR latencies and amplitudes [116,117]. Monitor the anesthesia. The duration of anesthesia depends on the species and the anesthetic used. For example, the duration of anesthesia induced by ketamine + xylazine is typically 30 to 45 min. After this time, half the dose may be administered as needed.

Do not leave an anesthetized animal unattended during the recovery process. Keep the animal warm by covering it. Return the animal to its cage as soon as it begins to move and allow it to recover fully. Record the recovery time in the log. Recording food and water consumption in the pre- and postoperative periods is good practice to confirm that animals are in a regular physical state after recovery from anesthesia. Intake of both will be reduced if the animal is in pain [98].

### 4.2. Performing ABR: Equipment

**Figure 4:**
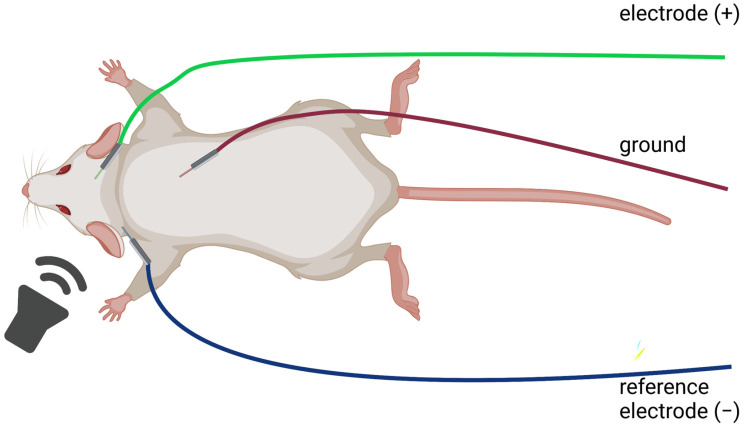
Example of electrode positioning for ABR. Created with BioRender.com.

Since significant amplitude variations can be related to electrode impedance, the impedance should be checked before ABR recording. Impedance >3 kΩ results in lower artifact suppression, lower recording quality, and incorrect threshold recognition at the sound pressure level of roughly 20 dB. Furthermore, the resistance between the recording and active electrodes should be tested. Since the placement of the reference electrode affects ABR recordings, carefully check the position of the needle electrodes [118]. A reference electrode is placed behind the ipsilateral ear. An active electrode is commonly placed on the vertex (base of the skull), and a ground electrode is placed at the back, hind hip, or base of the tail. Since the ABR is recorded from electrodes placed on the vertex and the electrode behind the ipsilateral ear [75], do not forget to change the reference electrode when switching between ears (the electrode’s position depends on the ear, which is measured; Figure 4).

The impact of the animal fat layer in the head/neck region on impedance has been previously discussed [24]. Since the ABR signal typically occurs in the 1–2.5 microVolt range, removing cables, wires, and noise generators from the test area is crucial.

Start the measurement. Correctly identifying the waveform, especially in the abnormal waveform, is challenging. Because waves II, IV, VI, and VII are inconsistent in humans, they are generally not considered for clinical interpretation [23]. To improve wave identification, an automated tool for ABR waves has been developed [119], which was possible because ABR has a predictable pattern. Therefore, time intervals for each wave were used to find the local extrema of the waveforms. The same rules are used to identify waves in animals. First, wave I is identified, and then the rest of the waves are identified. Depending on the literature, either wave II or III is the most prominent in rodents. The automatically detected ABR threshold is similar to the visually detected threshold [120].

At the end of the recording, remove the electrodes, disinfect them, and store them in a sterile container (record the number of times the electrodes were used in the protocol). Ensure that all recorded traces are saved for further analysis (.txt files can be uploaded into an Excel file) [70].

### 4.3. Performing ABR: Experiment

Any adverse events during the ABR recording should be noted in the protocol. The reasons for excluding an animal from the analysis should be recorded in the protocol. Room temperature variations should also be considered a confounding factor [121]. Detailed experimental records can help quickly identify the source of variability between animals, especially differences between animals during the same experiments [65]. A sex-specific analysis should be performed if male and female animals were included in the study. Data analysis should be based on the experimental design.

### 4.4. Protocols

The published research performed with ABR usually contains respective protocols. However, manuscripts focused on detailed ABR protocols have also been published in peer-reviewed journals, and we list them in Table 4 below.

## 5. Conclusions

ABR is a non-invasive technique that measures electrical potential reflecting neural activity in the auditory pathway and can be used to identify markers of different auditory conditions. Improving data reproducibility during preclinical studies is necessary to translate animal research into the clinic. Such improvement can be achieved by standardizing the design, conducting experiments, and reporting all information in publications according to the ARRIVE guidelines [125]. In addition, the management of sources of variability should be addressed in every publication [126], leading to a better understanding and improved translation of the results obtained and reducing the number of experimental animals used.

## Figures and Tables

**Table 1:** The onset of age-related hearing loss (ARHL or presbycusis) in selected mouse strains.

Mouse Strain	Onset of ARHL
C57Bl/6J	6 months [34]
CBA/J	20 months [32]
DBA/2J	3 weeks [35]
Balb/C	10 months [36]

**Table 3:** Advantages and disadvantages of using particular anesthetics for ABR studies.

Anesthetic	Sedation [73]	Drawbacks
Xylazine + Ketamine (i.p., i.m.)	Last ~45 min, the animal is awake after ~90 min from the initial injection. In male Wistar rats, complete sedation occurs in 10 min [89]	Requires proper restraining; rats anesthetized with this drug are more likely to develop corneal lesions than rats anesthetized with isoflurane, which is essential for long-term studies [90].
Isoflurane (inhalation)	Fast-acting, short-acting inhalation agent; the animal is usually fully sedated within 4–5 min. When the gas is removed, the animal wakes up very quickly.	Long-Evans rats anesthetized with isoflurane had higher hearing thresholds than rats anesthetized with ketamine/xylazine. Both click and tone thresholds were elevated, and the ABR response was worse [91,92].

**Table 4:** Publications focused on ABR protocols.

Title	Publication Year	Species	Ref.
Measurement of the auditory brainstem response (ABR) to study auditory sensitivity in mice	2006	mice	[122]
Using the Auditory Brainstem Response (ABR) to Determine Sensitivity of Hearing in Mutant Mice	2011	mice	[123]
Mouse Auditory Brainstem Response Testing	2016	mice	[114]
Data Acquisition and Analysis In Brainstem Evoked Response Audiometry In Mice	2019	mice	[72]
Protocol for assessing auditory brainstem response in mice using a four-channel recording system	2022	mice	[124]
Auditory brainstem response (ABR) measurements in small mammals,” in Developmental, Physiological, and Functional Neurobiology of the Inner Ear	2022	mice (suggested application also for rats, hamsters, and bats)	[71]

## Data Availability

No new data were created or analyzed in this study. Data sharing is not applicable to this article.
